# Salvage-Bestrahlung der Prostataloge: Mitbestrahlung der regionalen LK und Bedeutung der ADT

**DOI:** 10.1007/s00066-022-02001-5

**Published:** 2022-09-09

**Authors:** Felix Grabenbauer, Michael Flentje

**Affiliations:** Klinik und Poliklinik für Strahlentherapie, Uniklinikum Würzburg, Josef-Schneider-Str. 11, 97080 Würzburg, Deutschland

## Hintergrund

Die Salvage-Radiotherapie der Prostataloge („prostate bed radiotherapy“, PBRT) bei steigendem oder anhaltendem PSA-Wert nach Prostatvesikulektomie (PVE) führt bei 70 % der Patienten zu anhaltender Progressionsfreiheit nach 5 Jahren. Die SPPORT-Studie untersuchte in einer 3‑armigen Randomisierung, ob sich durch die Hinzunahme einer Androgendeprivationstherapie (ADT) über 4–6 Monate oder eine Bestrahlung der pelvinen Lymphabflüsse („pelvic lymph node radiotherapy“, PLNRT) mit ADT zusätzlich zur Standard-PBRT eine Verbesserung des progressionsfreien Überlebens erzielen ließe [1].

## Methoden

Aufgenommen wurden Patienten mit PSA-Persistenz oder steigenden PSA-Werten um 0,1–2,0 ng/ml nach PVE eines Adenoprostatakarzinoms (mit oder ohne Lymphknotendissektion). Entnommene Lymphknoten mussten tumorfrei sein. Weitere Einschlusskriterien waren eine pT2- oder pT3-Kategorie, ein Gleason-Score ≤ 9 und ein Zubrod Performance Status von 0 bis 1. Die Randomisierung erfolgte gleichgewichtet in drei Behandlungsarme: (1) Salvage-PBRT (1,8 Gy bis 64,8–70,2 Gy) vs. (2) PBRT plus Kurzzeit-ADT vs. (3) PBRT mit PLNRT (1,8–45 Gy auf regionale LK) plus Kurzzeit-ADT. Die ADT bestand aus einer Kombination von LHRH-Agonisten plus Flutamid oder Bicalutamid (50 mg oral/Tag) über 4–6 Monate vor RT bis zum Ende der RT. Primärer Endpunkt der Studie war die progressionsfreie Überlebensrate. Ein Ereignis wurde definiert als erstes Auftreten eines biochemischen Versagens gemäß der Phoenix-Klassifikation (PSA ≥ 2 ng/ml über dem Nadir-PSA), klinisch fassbares Rezidiv (lokales Versagen, regionale Metastasierung, Fernmetastasierung) oder Tod (jeglicher Ursache).

## Ergebnisse

Randomisiert wurden von 2008 bis 2015 insgesamt 1792 Patienten, ausgewertet wurden 1716 Patienten. Nach einer medianen Nachbeobachtungszeit von 8,2 Jahren (IQR 6,6–9,4) betrug die 5‑Jahres-Überlebensrate ohne Progression für die Patienten nach Standard-PBRT 70,9 % (95 %-CI 67,0–74,9), nach PBRT plus ADT 81,3 % (78,0–84,6) und nach PBRT mit PLNRT plus ADT 87,4 % (84,7–90,2). Damit war die zusätzliche Radiotherapie der pelvinen Lymphabflusswege in Kombination mit einer kurzen Androgendeprivation den beiden anderen Behandlungsarmen signifikant überlegen. In der Subgruppenanalyse betraf dies auch Patienten mit niedrigem PSA-Wert. Verbunden mit der Verbesserung der Progressionsfreiheit war eine signifikante Verlängerung der therapiefreien Zeit bis zur nächsten Salvage-Therapie. Das Gesamtüberleben war allerdings in allen drei Behandlungsgruppen praktisch identisch. Akute Toxizitäten (≤ 3 Monate nach der Strahlentherapie) von Grad 2 oder höher traten in Gruppe 3 signifikant häufiger auf (246 [44 %] von 563 Patienten) als in Gruppe 2 (201 [36 %] von 563; *p* = 0,0034), dort wiederum häufiger als in Gruppe 1 (98 [18 %] von 547; *p* < 0,0001). Die Spättoxizität (> 3 Monate nach der Strahlentherapie) unterschied sich nicht signifikant zwischen den Gruppen, lediglich in Gruppe 3 mit der Bestrahlung des Lymphabflussgebiets zeigten sich vermehrt hämatologische Nebenwirkungen von Grad 2 oder höher (*p* = 0,0060).

## Schlussfolgerung der Autoren

Die Daten bestätigen, dass vor allem die Kurzzeit-ADT zusätzlich zur Salvage-Bestrahlung der Prostataloge, aber auch die zusätzliche Bestrahlung der pelvinen Lymphabflusswege in Kombination mit einer ADT über 4–6 Monate eine bedeutsame Reduktion der Progressionsraten nach PSA-Rezidiv bewirken.

## Kommentar

In bisherigen Studien (GETUG, TROG, RADICALS) wurde im adjuvanten Setting nach PVE bereits der Vergleich bezüglich Effektivität und Sicherheit einer adjuvanten Radiotherapie vs. Observation mit ggf. Salvage-RT untersucht. Auf Grundlage der drei randomisierten, prospektiven Studien hat sich die frühe Salvage-Radiotherapie im Rezidiv gegen die adjuvante Bestrahlung durchgesetzt, was dem Vorgehen nach RADICALS-Studie entspricht [2].

Nach wie vor gibt es keine eindeutigen Empfehlungen bezüglich der Einbeziehung von Lymphknoten in die Salvage-Bestrahlungen, im Englischen „whole pelvic radiotherapy“ (WPRT). Schon 2018 zeigten Pollack et al. mit dieser Behandlungsmodalität eine Verbesserung des progressionsfreien Überlebens im Rahmen der SPPORT-Studie. Das Follow-up wurde fortgeführt, um die Unterschiede zwischen den Behandlungsarmen besser abbilden zu können. In der Studie wurde erstmalig die Salvage-Bestrahlung inklusive Erfassung der pelvinen Lymphknoten plus ADT prospektiv untersucht, angelehnt wurde das Design an die RTOG-9413-Studie, in der eine primäre WPRT mit neoadjuvanter AHT das PFS verlängerte, wenn auch nicht effektiver als in der Gruppe mit alleiniger Bestrahlung der Loge und adjuvanter AHT für 2 Jahre [3].

Die Studienqualität scheint gesichert. 87 % der Patienten erhielten in der SPPORT-Studie eine IMRT und die Bestrahlungsdaten und Pläne zeigten bei zentraler Prüfung eine hohe Protokolltreue. Eine Besonderheit ist die Anwendung der Phönix-Kriterien (Therapieversagen bei PSA > 2 ng/ml über Nadir) auch beim PSA-Rezidiv nach PVE. Die Autoren begründen dies im Vergleich zu niedrigeren Schwellen (gebräuchlich 0,4 oder 0,5 ng/ml) mit der besseren Korrelation zu klinischen Endpunkten.

Die beste Rate an progressionsfreiem Überleben nach fünf Jahren erreichte in der SPPORT-Studie der Studienarm mit Erweiterung des Bestrahlungsfelds um das Gebiet der pelvinen Lymphknoten mit zusätzlicher (neoadjuvanter) Kurzzeit-ADT. Anhand der Follow-up-Daten mit einer Nachbeobachtungszeit von 8 Jahren zeigt sich vergleichend zwischen Gruppe 2 (PBRT + ADT) und Gruppe 3 (PBRT + PLNRT + ADT) ein Rückgang der Signifikanz von *p* < 0,00027 (PFS 81,3 % vs. 87,4 %; 5 Jahre) auf *p* < 0,048 (PFS 71,8 % vs. 76,9 %; 8 Jahre), womit ein gewisser Rückgang des Effekts in größerem Zeitabstand abgebildet wird.

Die Bestrahlung der Prostataloge erfolgte hier im Median mit 68,4 Gy (ED 1,8 Gy). Bei Vorgabe der Bestrahlungsdosis von 64,8 bis 70,2 Gy berufen sich die Untersucher auf einen positiven Dose-response-Effekt unter Dosiseskalation [4]. Dem gegenüber stehen Daten der zweiarmigen SAKK-09/10-Studie, in der sich die Behandlungsarme mit 64 Gy oder 70 Gy (ED 2 Gy) nicht in progressionsfreiem Überleben unterschieden [5]. Allerdings war die SAKK-Studie gepowert für ein Low-risk-Patientengut mit kleinem High-risk-Kollektiv, wohingegen in einer Studie aus Peking von Qi et al. ein positiver Effekt für die Dosiseskalation mit 72 Gy bei Gleason 8+ gezeigt wurde [6]. Zumindest in der Low-risk-Gruppe ist eine weitere (konventionelle) Dosiseskalation im Tumorbett fraglich.

Eine weitere Überlegung in diesem Setting ist die gezielte hoch dosierte/hypofraktionierte Bestrahlung an Prostata oder Lymphknoten. Für eine hypofraktionierte Bestrahlung der Prostata spricht die Beziehung der α/β-Werte von Zielstruktur und umliegender Gewebe bei α/β-Werten der Prostatatumoren von 1,5 Gy und umliegender Organe wie Urethra, Blase oder Rektum von 3 bis 6 Gy. Gründe, die gegen die Hypofraktionierung sprechen, sind eine mögliche Steigerung der Nebenwirkungen, insbesondere an neurovaskulärem Bündel (sofern verblieben) und vesikourethraler Anastomose. Daten zur stereotaktischen Salvage-Bestrahlung sind derzeit noch unterrepräsentiert, zumal die Qualität der Daten noch nicht für eine richtungsweisende Aussage ausreicht [7].

Da durch die Bestrahlung der Lymphknoten mit 45 Gy eine gesteigerte Rate an progressionsfreiem Überleben erlangt wird, die aber langfristig tendenziell an Effekt zu verlieren scheint, ist zu erwägen, dass die applizierte Dosis teilweise keinen nachhaltigen Effekt auf befallene Lymphknotenstationen hat. Das Staging vor Studieneinschluss umfasste entsprechend dem Rekrutierungszeitraum ein CT von Abdomen/Becken und eine Tc-99m-Skelettszintigraphie, die Methode der PSMA-PET-CT war zu dieser Zeit nicht ausreichend etabliert. Damit sind die heutigen Möglichkeiten zur Anpassung von Dosis (SIB) und Zielvolumen [8] sowie zur Stratifizierung von Risikogruppen [9] nicht eingegangen. Ein Einbeziehen der PSMA-Diagnostik könnte ein nächster Schritt zur Verbesserung der Salvage-Therapie sein. Für eine Kurzzeitandrogenblockade liefern die Daten der SPPORT-Studie und der vorhandenen übrigen Studien jedoch Unterstützung, da die absolute Senkung der Progressionsrate die Notwendigkeit einer intensiveren AHT bei Sekundärprogress signifikant reduziert. Möglicherweise ist es ein valider Behandlungsansatz, zumindest den Lymphabfluss erst im Falle eines erneuten Rezidivs nach PET-Bildgebung intensiviert zu bestrahlen.

## Fazit

Sowohl die Kurzzeit-ADT als auch (in geringerem Maße) die RT der pelvinen regionalen Lymphknoten verbessern die Progressionsfreiheit bei Salvage-RT wegen PSA-Anstieg. Für Patienten bedeutet dies zwar keinen Überlebensvorteil, aber eine wesentlich längere therapiefreie Zeit bis zur nächsten Salvage-Therapie. Diese Vorteile sind gegen die leicht erhöhte Rate an Nebenwirkungen abzuwägen. Die aktuelle S3-Leitlinie von Oktober 2021 empfiehlt, dass Patienten mit Indikation zur Salvage-RT bei einem PSA > 0,7 ng/ml eine zusätzliche ADT angeboten werden soll. Die SPPORT-Studie bestätigt letztendlich diese Empfehlung und liefert weitere Informationen, die für eine Beratung der Patienten im Sinne eines „shared decision-making“ hilfreich sind. Offen bleibt allerdings weiterhin die Frage, welche Bedeutung das Staging mittels PSMA-PET in der Primär- und Rezidivdiagnostik für die Therapieentscheidung bei Salvage-RT hat.

*Felix Grabenbauer, Michael Flentje, Würzburg*
